# Exercise improves the social and behavioral skills of children and adolescent with autism spectrum disorders

**DOI:** 10.3389/fpsyt.2022.1027799

**Published:** 2022-12-22

**Authors:** Chrystiane V. A. Toscano, José P. Ferreira, Ricardo T. Quinaud, Keity M. N. Silva, Humberto M. Carvalho, Joana M. Gaspar

**Affiliations:** ^1^Institute of Physical Education and Sport, Federal University of Alagoas, Maceió, Alagoas, Brazil; ^2^Faculty of Sport Sciences and Physical Education, University of Coimbra, Coimbra, Portugal; ^3^Department of Physical Education, School of Sports, Federal University of Santa Catarina, Florianópolis, Santa Catarina, Brazil; ^4^Physical Education Service, Unified Center for Integration and Development of Autism, Maceió, Alagoas, Brazil; ^5^Graduate Program in Biochemistry, School of Biological Sciences, Federal University of Santa Catarina, Florianópolis, Santa Catarina, Brazil

**Keywords:** autism, physical activity, interaction, stereotypes, Bayesian analysis

## Abstract

**Background:**

Currently, there is no standard treatment for Autism Spectrum Disorders (ASD), but there are many ways to minimize the symptoms and maximize abilities. Some studies suggest that exercise and other physical activities with children with ASD may be beneficial. In this study, we hypothesized that a physical exercise program (48-week exercise-intervention) could improve symptomatology dyad among children and adolescents with ASD. Our main aim was to examine the effects of physical activity on the primary clinical symptoms and associated comorbidities in children and adolescents with ASD.

**Methods:**

We allocated 229 children with ASD, ranging in age from 2.3–17.3 years (*M* = 7.8, *SD* = 3.2), into three groups: (a) exercise- intervention group, (b) control group from the same institution, and (c) control group from another institution. The exercise program was performed at moderate intensity in a 30 min section twice a week for 48 weeks. We used Bayesian multilevel regression modeling to examine participant outcomes and responses to the exercise-intervention.

**Results:**

Our results showed that a 48-week exercise-intervention substantially decreased ASD social interaction problems, attention deficit, emotional reactivity, stereotypical verbal and motor behavior, and sleep disturbances. However, physical exercise did not affect eye contact and food selectivity. We also observed that ASD severity and socioeconomic status influence eye contact, attention deficit, and sleep disturbance responses.

**Conclusion:**

In conclusion, children and adolescents with ASD exposed to a 48-week physical exercise-intervention program had important improvements in ASD symptoms. This study highlights that structured exercise programs can be a powerful complementary therapy for the ASD population.

## Introduction

Autism Spectrum Disorder (ASD) is characterized by: (a) Persistent deficits in social communication and social interaction; and (b) restricted and repetitive patterns of behavior, interests, or activities (1). An ASD diagnosis typically requires information from observers (such as parents and teachers) and observations of these symptoms in different social contexts (2–4). The National Health Center for Health Statistics has estimated that, in 2016, ASD had a prevalence of 1 in 36 children (5), and many have observed a higher ratio of boys to girls (e.g., 4:1) (6). ASD can be classified into three levels of severity: (a) level 1, involving mild autism and requiring support; (b) level 2, requiring substantial support; and (c) level 3, involving the most severe form of autism and requiring very significant support (1). However, the severity of social skills and behaviors can be influenced by differences in the environment and an individual child’s growth and development (7). In addition, children with ASD have a higher risk of comorbidities than children with normal neurodevelopmental (8–10). These comorbidities (motor deficits, obesity, sleep disorders, and gastrointestinal dysfunction) may potentiate main ASD social and behavioral symptoms.

Presently, no pharmacological treatments are available to cure autism. However, ASD treatment requires pedagogical, psychotherapeutic, and pharmacological interventions to control specific behavioral symptoms. ASD subjects engage in less physical activity than typically developing peers. Studies have proposed that limited levels of physical activity and late motor skills and fitness, particularly in children and adolescents with ASD, may accentuate social and emotional deficits and the associated comorbidities. This can result in serious negative consequences for individual wellbeing and may contribute to the physical, behavioral, and emotional challenges associated with ASD.

Studies have been demonstrated that physical exercise ameliorates deficits in social interaction (11), reduces aggressive behaviors (11–13), and reduces stereotypical behavior in children, teenagers, and adults with ASD (14–16). Recently, it was demonstrated that combined physical training improves restricted and repetitive patterns of behavior and social skills of ASD children (17, 18). In addition, physical exercise has a positive influence on different symptomatology and comorbidities, such as physical motor deficit reduction (19). However, it is also necessary to consider that the exercise-intervention studies described above included limited sample sizes and do not guarantee data on the impact of exercise-interventions on complete ASD symptomatology (clinical symptoms and comorbidities) and severity.

This study hypothesized that a 48-week physical exercise program could improve symptomatology profile and comorbidities among children and adolescents with ASD. This study aimed to examine the effects of an exercise program on the clinical symptoms in children and adolescents with ASD. Our study contributes with a large sample and considers the potential influence of children and adolescents’ characteristics at baseline, gender, age group, ASD severity, medication, and socioeconomic status.

## Materials and methods

### Study design and participants

This was a non-randomized controlled trial following the Guidelines for Reporting Non-Randomized Studies (20) and following the ethical standards of the World Medical Association Declaration of Helsinki. Therefore, the study was approved by the Federal University of Alagoas Ethical Committee (CAAE: 41286815.0.0000.5013) and by the Federal University of Santa Catarina Ethical Committee (CAAE: 82587818.3.0000.0121). In addition, all study participants and their families received information about the study protocol and signed an informed consent form (or child assent for younger children).

Participant inclusion criteria were a diagnosis of ASD based on DSM-5 criteria (1). The children and adolescents were heterogeneous regarding clinical symptoms and psychotropic medication use. The diagnoses of ASD were established by an experienced psychiatrist based on DSM-5 guidelines. All study participants were monitored by a multidisciplinary team that performed, every 6 months, the application of the Childhood Autism Rating Scale (CARS) and Autistic Traits Assessment Scale (ATA) to monitor the diagnostic stability of all participants during the study protocol. The level of support necessary for each participant was confirmed by the clinical psychiatrist and a multidisciplinary team of the institution, following the criteria established in the DSM-5. During the entire follow-up of the study, none of the participants presented means lower than the cut-off point shown in the ATA and CARS scales.

We considered participants for analysis by their ASD level of support as follows: level 1 (*n* = 143), level 2 (*n* = 63), and level 3 (*n* = 23). According to the DSM-5, the degrees of autism spectrum disorder vary according to the fundamental characteristics of the condition, which are deficits in communication and social interaction and restrictive and repetitive patterns.

**FIGURE 1:**
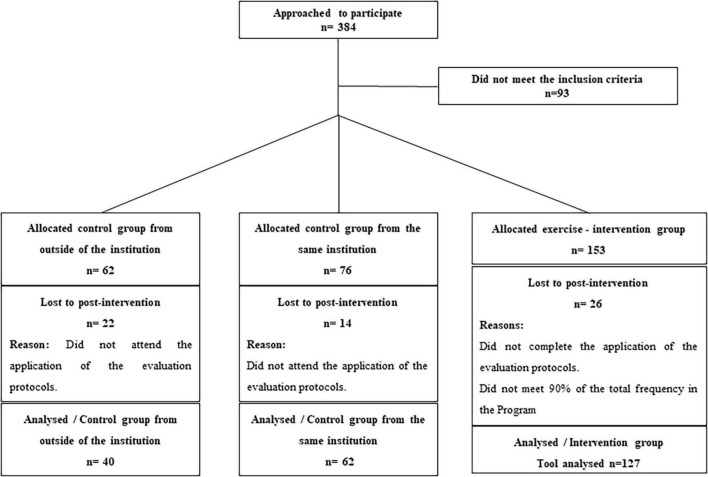
A controlled trial of the effects of a physical exercise program on the symptom profile of children and adolescents with ASD. Error bars indicate the 95% credible intervals.

A total of 229 children with ASD, recruited from a pediatric center for populations with ASD located in Maceió/Alagoas, Brazil, were considered for their data analysis (Figure 1). Of these, 33 were female, and 196 were male. Patients without a complete diagnosis of ASD (81 individuals) were excluded from the study, and children diagnosed with Rett’s syndrome (12 individuals). Children diagnosed with Rett’s syndrome were excluded based on the recommendations established by the DSM-5, even considering possible etiological similarities with autism (1). In addition, we considered an intervention group [*n* = 127, age 8.3 (3.3) years] and a control group from the same institution [*n* = 62, age 8.5 (3.2) years]. All participants and their families willing to be engaged in the exercise program were allocated to the exercise-intervention group. Those not interested in participating in the exercise program were assigned to the control group. Also, we considered a second control group composed of age-matched children with ASD from a different institution in Maceió/Alagoas [*n* = 40, age 7.4 (2.8) years]. The second control group allows us to account for potential environmental variation in the outcomes.

### Procedures and exercise-intervention program

The intervention group was exposed to a 48-week physical exercise program designed specifically for the population with ASD (21). According to previous studies (16, 22, 23), adaptive procedures adjustments are needed to apply physical exercise programs to children and adolescents with ASD. In addition, given the heterogeneity of the clinical characteristics of the participants (indicated by the application of scales such as the ATA, as well as the indication of the level of support), physical exercise procedures need to be adjusted according to each level of support for each specific child. Therefore, during the 4-week adaptation phase to the Physical Exercise Program, their family mediator accompanied children and adolescents in physical exercise sessions. The main role of the mediator was to facilitate communication and interaction between the participant with ASD and the Physical Education teacher in the process of carrying out the exercises proposed in the program.

During the adaptation sessions, each participant’s individual sheet was recorded: (i) Items from the Autistic Traits Scale considered indicators of a better profile for receiving the information offered by the mediator. For example, the items “difficulty in social interaction” was identified; “environmental manipulation,”; “resistance to change,”; “lack of eye contact,”; “lack of attention,” and “hyperactivity/hypoactivity” and “stereotyped and repetitive movements.” In addition, dangerous problem behaviors were also recorded (physical aggression and self-injurious behaviors), and (ii) the presence of risk behaviors and identification of possible environmental causes (the characteristics of the program, the environment, the group of children or adolescents belonging to the activities, and the mediator of the activities).

Interviews were conducted with each family mediator to identify preference items (stimuli that the person prefers) to be used as reinforcement during physical exercise sessions when necessary. The checklist used at the Center for ASD at the University of Nebraska Medical Center was used, which contains items such as toys, activities, electronics, and other things. The family member must indicate to the interviewer whether or not the item is a favorite item for their child. Each interview lasted 15–20 min and was carried out with the specialized center. After identifying the items, they were tested during adaptation sessions and, if confirmed, were used during physical exercise sessions to increase the child’s or adolescent’s engagement time in the exercise session (21, 22). In addition, family members were interviewed, questions were related to a list of 38 items distributed in the category’s toys, activities, and electronics, and an open question aimed at recording other items of preference for the child not listed in the categories mentioned above. The purpose of using the checklist to identify preference items in this study was to define the stimuli that the mediators of the exercise-intervention could use as reinforcers for better engagement and permanence of the child or adolescents in the program sessions.

After the adaptation phase, 40 weeks of interventions were carried out. The intervention program was composed of sessions of basic coordination and strength exercises, consuming 40 min per session, with sessions occurring twice a week and totaling 96 over the observation period. All intervention sessions were directed by a physical educator with experience working with children with ASD (16, 21). The sessions were held at the facilities of the Pediatric Center specialized in the population with ASD located in Maceió, Alagoas, Brazil. The physical exercise-intervention sessions had the following structure: (i) Preparatory phase for the exercise session (5 min); (ii) development phase (30 min) in which children performed a brief warm-up and then performed strength, balance, and coordination exercises; and (iii) return to calm phase (5 min), in which parents and legal representatives assisted with the relaxation exercises using tactile slip skills on the child’s back and belly aiming to return the child to calm. The development phase’s coordination, strength, and balance activities included climbing and support in a bar, adapted basketball shot, elastic bands, workout, climbing the steps and walking on the inclined plane, step box with a target, and sequenced march. A description of the exercises is provided as supplementary material,^1^ and further details about the exercise program design, contends, and settings were previously published (21, 22).

The first control group participants were exposed to the same care within the specialized pediatric center for populations with ASD as the intervention group. Still, they did not participate in the intervention exercise sessions. Therefore, only participants who attended at least 90% of the total sessions across the observation period were considered for data analysis. The care for ASD children in specialized pediatric centers is standardized within the national health system and regulated by the Brazilian Ministry of Health (24).

Therefore, we also considered including an independent control group from another institution that followed the same healthcare standards defined by the Brazilian ministry of health to match and adjust the participants with children with ASD from the same and another institution to account for potential environmental variation in the outcomes.

### Outcome measures

**Autistic traits assessment scale:** This is a protocol for identifying the symptoms of the autism spectrum disorder and is also used to examine the evolution of ASD characteristics. It is an easy-to-use protocol, accessible to non-clinical professionals who know the condition. The ATA scale consists of 23 subscales, each with criteria items of ASD characteristics. To explore the characteristics that integrate the ASD symptomatology and that directly interfered in the elaboration of the intervention model with physical exercise in this study, we considered the following eight subscales for analysis: Social interaction (7 items); eye contact (6 items); attention deficit (6 items); reactivity (8 items); motor stereotype (7 items); and verbal stereotype (8 items); sleep disturbance (5 items); food selection (8 items). We used the Brazilian Portuguese version, translated and validated after adjustments to the diagnostic criteria following the DSM-IV guidelines (25). For the six subscales, criteria items are identified and counted in each subscale. The scale displayed adequate internal consistency reliability (α = 0.71) (25).

**Childhood autism rating scale:** This is a standardized instrument to identify levels of intensity of ASD (mild, moderate, and severe), as well as the sharp distinction between autism and intellectual disability (26). The Brazilian Portuguese version of this instrument (CARS-BR) (26) was used to evaluate the child’s behavior from 14 domains usually affected by serious problems in autism, plus a general category of autism impressions. The 15 items of the scale are relative to people, imitative behavior, emotional response, body use, use of an object, adaptation to change, visual response, auditory response, perceptual response, fear or anxiety, verbal communication, non-verbal communication, activity level, level and consistency of intellectual relations, and general impressions. The validation process demonstrated a very good internal consistency, with mean values of Cronbach’s alpha coefficient of 0.82 (95% CI, 0.71–0.88), indicating a high degree of internal consistency (26). Compared to the ATA scale, data on the convergent validity showed Pearson’s correlation coefficient values with *r* = 0.89. The test-retest reliability had a kappa coefficient value of 0.90.

**Anthropometric measurements:** A single experienced observer performed anthropometric measurements. Each participant was measured individually, in a private room at the pediatric center, in the presence of the parents, following standardized procedures (27). When necessary, adaptive approaches were prepared previously for the assessment to meet the standardized methods for anthropometric measurements (using the tablet, cell phone, and toys as a reflector of adaptive behavior) (21, 23). Stature was measured with a portable stadiometer (Seca model 206, Hanover, MD, USA) to the nearest 0.1 cm. Body mass was measured with a calibrated portable balance (Seca model 770, Birmingham, UK) to the nearest 0.1 kg. Waist circumference was measured with an anthropometric tape (Seca model 201, Birmingham, UK) to the nearest 0.1 cm.

**Chronological age:** Chronological age was calculated to the nearest 0.1 year as birth date minus testing date. At baseline, participants were aged 7.8 (3.2) years, on average, ranging between 2.3 and 17.3 years. Given the range in the sample, we grouped participants by age, assuming the World Health Organization as a reference (28): 0–4 years (*n* = 51); 5–9 years (*n* = 126); 10–14 years (*n* = 47); and 15–19 years (*n* = 5).

**Socioeconomic status:** The application of the questionnaires to the parents was carried out before the session by the researcher. First, we applied a questionnaire for the parents to establish socioeconomic status developed by the Brazilian Association of Market Research Institutes (29). The questionnaire allows participants to be grouped into eight categories, where A1 represents the highest and E the lowest. The categories are calculated based on the average gross monthly income of the families, according to standardized or potential consumption patterns, and the classification of the level of education of the family supporter (from illiteracy to higher education level).

**Medication:** Information about the psychotropic medication (antiepileptics, antipsychotics, antidepressants, and stimulants) used by the participants was obtained from medical records. However, access to children’s proportions of the medication doses prescribed was not available. Therefore, we grouped participants by medications as follows: No prescription (*n* = 51); medicated only with antidepressants (*n* = 7); medicated only with antiepileptics (*n* = 9); medicated only with antipsychotics (*n* = 3); medicated only with stimulants (*n* = 93); medicated with antiepileptics and stimulants (*n* = 12); medicated with antidepressants and stimulants (*n* = 33); medicated with antipsychotics and antiepileptics (*n* = 1); medicated with antipsychotics and stimulants (*n* = 6); medicated with antidepressants, antiepileptics, and stimulants (*n* = 13); medicated with antipsychotics, antiepileptics and antidepressants (*n* = 1).

### Data analysis

We fitted multilevel ordinal models in a fully Bayesian framework (30, 31) to examine the effects of an exercise program on symptomatology among children and adolescents with ASD. Multilevel models allow and explicitly model the data structure by allowing for residual components at each level in the hierarchy or cluster (32). In addition, multilevel models partially pool the information across units to produce better estimates for all units in the data (33), proving an approach to minimize the selection bias of the design. Hence, we used varying intercepts, varying slope models to adjust for differences in both time-invariant and time-variant characteristics of the sample (34). The changes in the accumulated number of items by a subscale of the ATA scale for each participant were modeled as function exposure to intervention or control groups (i.e., exercise-intervention group, control group from the same institution, or control group from another institution) as population-level effects, and adjusting for the influence of ASD severity level, gender, medication, age group, and socioeconomic status as group-level effects. Hence, we explicitly modeled participants’ characteristics in the multilevel models to account for potential uncertainty bias (35). We allowed changes across the 48 weeks to vary by participants and the intercepts to vary by cross-classified groups.

The ordinal models were fitted with sequential models assuming the same effect on all response categories (30). Our estimations were regularized using weakly informative prior distributions, normal prior (0, 10) for population-level effects, and normal priors (0, 1) for group-level effects. We run four chains for 4,000 iterations with a warm-up length of 1,000 iterations to ensure convergence of the Markov chain. To check the convergence of Markov chains, we inspected trace plots and posterior predictive checks to validate our models (31). The Bayesian methods were fitted using the brms package (30), available in the R statistical language (36), which calls Stan (37), to compute the posteriors. To ensure the transparency and reproducibility of our analyses, a public repository containing all the data and code needed to replicate the analyses and figures is available at: https://osf.io/v6jyx/.

## Results

The characteristics of children with ASD at baseline in the study are summarized in Table 1. The Bayesian methods used in the analysis allow direct probabilistic interpretations of credible (also called confidence intervals) and posterior probabilities (38).

**TABLE 1:** Baseline characteristics of children with autism spectrum disorder grouped as intervention and control groups.

	Exercise-intervention group (*n* = 127)	Control group same institution (*n* = 62)	Control group another institution (*n* = 40)
Chronological age (years)	7.9 (3.3)	8.1 (3.2)	6.9 (2.8)
Stature (cm)	127.4 (14.9)	125.5 (18.1)	132.4 (27.4)
Body mass (kg)	34.9 (14.9)	33.4 (14.3)	41.9 (24.0)
Body mass index (kg/m^2^)	21.0 (6.8)	20.9 (7.9)	22.8 (8.1)
Social interaction (0–7)	5.2 (1.7)	5.6 (1.6)	4.0 (1.5)
Eye contact (0–6)	5.2 (1.4)	5.2 (1.5)	4.8 (1.2)
Attention deficit (0–6)	4.1 (1.1)	4.1 (1.0)	3.6 (0.9)
Verbal stereotype (0–8)	4.6 (1.3)	4.6 (1.6)	4.9 (1.4)
Reactivity (0–8)	4.1 (1.1)	3.9 (1.2)	4.3 (1.2)
Motor stereotype (0–8)	5.0 (1.7)	4.9 (1.9)	6.3 (1.4)
Sleep disturbance (0–5)	2.5 (1.2)	3.8 (1.4)	3.7 (1.3)
Food selection (0–8)	3.6 (1.4)	3.5 (1.5)	3.3 (1.4)

**FIGURE 2:**
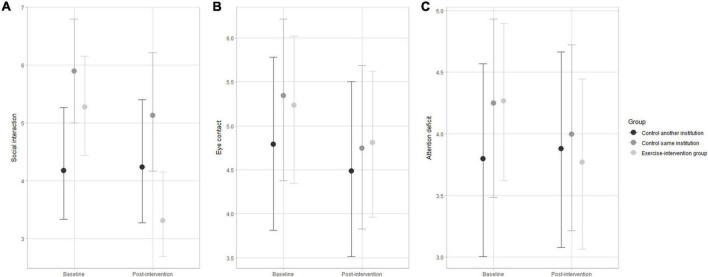
Changes in social interaction **(A)**, eye contact **(B)**, and attention deficit **(C)** pre- and post-intervention in the exercise-intervention, same institution control, and another institution control groups. Error bars indicate the 95% credible intervals.

**FIGURE 3:**
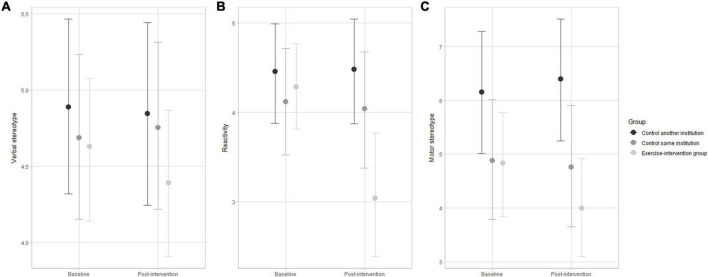
Changes in verbal stereotype **(A)**, reactivity **(B)**, and motor stereotype **(C)** pre- and post-intervention in the exercise-intervention, same institution control, and another institution control groups. Error bars indicate the 95% credible intervals.

**FIGURE 4:**
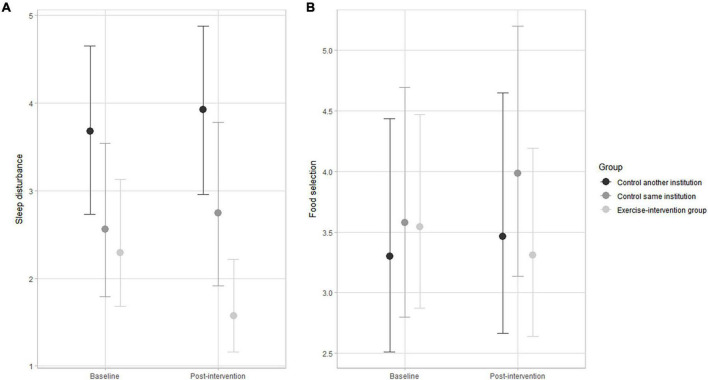
Changes in sleep disturbance **(A)** and food selectivity **(B)** pre-and post-intervention in the exercise-intervention, same institution control, and another institution control groups. Error bars indicate the 95% credible intervals.

Multilevel ordinal modeling results and respective 95% credible intervals of changes across the 48-week exercise-based intervention for the eight autistic traits are displayed in Figures 2–4. The subscale scores pre- and post-intervention were adjusted for ASD severity level, gender, medication, age group, and socioeconomic status as group-level effects. The overlap of the 95% credible intervals at baseline implies baseline variation between participants from the intervention group and control groups for social interaction (Figure 2A), motor stereotype (Figure 3C), and sleep disturbance (Figure 4A) subscales. At baseline, the participants displayed a similar profile of autistic symptoms for the other subscales.

The exercise-intervention group showed substantial decreases in the accumulated number of items for social interaction after 48 weeks compared to both control groups (Figure 2). There was a small decrease in items after 48 weeks for eye contact and attention deficit, but uncertainty estimates were large for all groups. The variation in the accumulated number of items for eye contact and attention deficit was substantially influenced by ASD severity level and socioeconomic status.

For reactivity, the exercise-intervention group displayed a substantial decrease in the accumulated number of items after the 48-weeks intervention (Figure 3B) compared to both control groups. Verbal and motor stereotypes showed a trend of decline in the accumulated number of items to the exercise-intervention, as there was no change for both control groups. Nevertheless, the interpretation must be conservative, given the large uncertainty estimates. The variation in the accumulated number of items for verbal stereotypes was substantially influenced by socioeconomic status.

The exercise-intervention group showed substantial decreases in the accumulated number of items of sleep disturbance after 48 weeks compared to the slight trend of increase observed in both control groups (Figure 4A). As for food selectivity, the exercise-intervention group showed no substantial change after 48 weeks, in contrast with the trend of an increase in the number of items in the subscale in the control groups, particularly for the group from the same institution (Figure 4B). The variation in the accumulated number of items for sleep disturbance and food selectivity was substantially influenced by ASD severity level and socioeconomic status.

## Discussion

In this study, we demonstrated practice of physical activity by children and adolescents with ASD ameliorates social interaction, motor stereotypes, sleep disturbances, and for reactivity. No changes were observed for verbal and motor stereotypes, as well as for food selectivity. We also observed that the variation for eye contact, attention deficit, verbal stereotypes, sleep disturbance, and food selectivity was substantially influenced by ASD severity level and socioeconomic status. Our study demonstrates that 48 weeks of exercise-intervention substantially improved the two domain structures. Physical exercise-interventions have shown positive effects in reducing core symptoms of ASD and associated clinical comorbidities (11, 12, 14, 22, 23, 39–41).

However, available data are limited by small sample sizes, warranting data on the impact of exercise-interventions on ASD symptomatologic profile and severity. Our study contributed with a large sample and considered the potential influence of children and adolescents’ characteristics at baseline, gender, age group, ASD severity, medication, socioeconomic status, and specialized pediatric institution. To our best knowledge, the study of the effects of intervention with exercise on the social interaction of children and adolescents with ASD is sparse. Nevertheless, our observations showed a substantial reduction in the number of items for social interaction and attention deficit after the 48-week intervention for the exercise-intervention group compared to the control group in the same institution and another institution. Our results are consistent with observations showing positive social intervention time and reduced social dysfunction in children with ASD when exposed to Kata techniques training (42).

On the other hand, children with ASD exposed to aquatic exercise showed positive sensory benefits, decreasing maladaptive behaviors. Still, there was no identification of increased social skills needed for interaction (43). Different methodological approaches across the available studies may likely explain some inconsistencies. It is difficult to define instruments and their reliability to assess the effects of exercise on the social interaction of children with ASD (43, 44). Importantly, improvements in autistic social traits could predate potential benefits in eating disorder behaviors (45).

The repetitive and restricted patterns of behaviors, also referred to as stereotypical behaviors, deserve special attention because they produce losses in the motor profile and children’s interaction with the environment (46, 47). In the present study, the patterns of behavior of children and adolescents with ASD (i.e., reactivity, motor, and verbal stereotypes) exposed to the 48-weeks intervention showed a substantial improvement compared to controls. Our results are consistent with observations noting a reduction of stereotyped motor behaviors and reactivity with exercise exposure. Available data in the literature mainly includes intervention models based on aerobic exercises (running, cycling, and ball games) (13, 48–50), combined exercises (22, 51), and exercises Kata technique (42, 52).

Sleep disorders are presented in about 50–80% of children and adolescents with ASD (53, 54) and are likely to have more severe primary symptoms (55, 56). In addition, fragile X syndrome (FXS), the leading cause of inherited autism spectrum disorder, is associated with multiple neurobehavioral abnormalities, including sleep difficulties related to irritability/aggression and hyperactivity (57). Given the high prevalence of sleep disorders in the population, it has been recommended to explore the effects of intervention with exercise on possible improvements in sleep quality and the increase in the total number of hours of sleep for the ASD population (58). In the present study, the children and adolescents exposed to the 48 weeks of exercise-intervention had substantial improvements in sleep disturbance. In contrast, the participants in both control groups show a trend of an increase in sleep disturbances. Hence, our observations confirm the suggestion that clinical professionals should prescribe intervention with physical exercise for children with ASD, especially for those with sleep disorders and low adherence to using medications (53, 59). Furthermore, the positive influence of exercise on sleep quality and quantity may be mediated by the impact of exercise on pathophysiological mechanisms, mainly in increasing their endogenous melatonin levels (53).

Eating disorders are presented in about 45–95% of children and adolescents with ASD (55, 60) and are associated with more severe primary symptoms (55). In addition, children and adolescents with ASD have an increased risk of obesity and obesity-related metabolic disorders (57, 61, 62). However, to our best knowledge, no observations are available on exercise-interventions’ effects on eating disorders’ symptoms. The children and adolescents with ASD exposed to the 48-week intervention in this study showed improved eating disorders. Previously, we observed an improvement in cholesterol (increase in HDL-C, decrease in LDL, and total cholesterol) in response to exposure to the 48-week exercise-intervention (22). On the other hand, it has been noted that autistic social behavior appears to predate eating disorder behaviors (46). Hence, the positive effect of exercise on social interactions may positively influence eating disorders behaviors, likely contributing to improving the cholesterol profile of children and adolescents with ASD.

It should be noted that the ATA scale is based on information about children’s symptoms provided by their parents. Parents were trained to identify behavior changes in their children, and the intervention program coordinator assisted the families when necessary. The ATA scale is one of the few instruments validated and translated for the Brazilian population (25). In addition, the protocol provides an easy-to-use protocol available to non-clinical professionals with knowledge of the condition. Given the available sample size, design, and the applied use of the method, we assume the value of the ATA scale to explore the characteristics that integrate the ASD symptomatology profile and that directly interfered in the elaboration of the intervention model with physical exercise.

## Conclusion

In conclusion, this study demonstrates that the 48-week exercise-intervention improves the social and behavioral skills of children and adolescents with autism spectrum disorders, confirming the study hypothesis. Therefore, highly structured and personalized exercise programs should be a powerful complementary therapy to minimize symptomatology among children and adolescents with ASD.

### Study limitations

The present study’s main limitation lies in using a non-randomized study design, which is more susceptible to bias. The main sources of bias in non-randomized study designs include (a) selection bias, i.e., systematic differences between comparison groups; (b) performance bias, i.e., systematic differences in the care provided apart from the intervention being evaluated; (c) detection bias, i.e., systematic differences in the assessment of outcome(s), and (d) attrition bias systematic differences in withdrawal from the study (20, 35).

To minimize bias and given that allocation to the exercise-intervention group was made by participant (parent) choice, we explicitly adjusted our models to individual characteristics, including gender, age, ASD severity, medication, socioeconomic status, and contextual aspects of the healthcare provider. In particular, we considered including an independent control group from another institution that followed the same healthcare standards defined by the Brazilian ministry of health to match and adjust the participants with children with ASD from the same and another institution to account for potential environmental variation in the outcomes.

Lastly, appropriate statistical modeling allows us to minimize sources of bias in non-randomized study designs (35). We used a Bayesian multilevel modeling approach to deal with the study design and the data structure. Bayesian methods provide a natural approach to account for different sources of inferential uncertainty (33, 63–65). The parameters are treated as random variables combining both sample data and prior distribution information to estimate a (posterior) probability distribution that reflects the uncertainty associated with how well they are known based on the data (33, 65).

Intuitively, the repeated measures data present either crossed or nested levels, where effects are likely to vary between different sub-groups (66). Traditional single-level analyses (e.g., analysis of variance and co-variance) are often used, although inappropriately (31, 33, 66). The multilevel modeling framework allows individual changes in the outcomes of interest to be modeled as a function of individuals, group allocation, and contextual covariates, providing aggregated estimates of a target group and significantly improving estimations of small and sparse group data (34). Another limitation of our study is that the majority of the sample had ASD children with support level 1 [mild ASD (62%)], and there is also a lack of data on syndromic ASD, such as FXS (56, 57).

## Data availability statement

The raw data supporting the conclusions of this article will be made available by the authors, without undue reservation.

## Ethics statement

The studies involving human participants were reviewed and approved by the Federal University of Alagoas Ethical Committee (CAAE: 41286815.0.0000.5013) and Federal University of Santa Catarina Ethical Committee (CAAE: 82587818.3.0000.0121). Written informed consent to participate in this study was provided by the participants’ legal guardian/next of kin.

## Author contributions

CT and JG designed and drafted the manuscript. RQ and KS helped with intervention program and data collection. HC and JF analyzed, interpreted data, and revised critically the manuscript. All authors contributed to the article and approved the submitted version.

## Footnotes

1
https://osf.io/v6jyx/
